# Choosing courtship over caution: male jumping spiders delay anti-predator responses

**DOI:** 10.1186/s12983-026-00604-7

**Published:** 2026-04-01

**Authors:** Miriam Scriba, Jessy Auerswald, Linnea Lehnen, Jutta M. Schneider

**Affiliations:** Fakultät Für Mathematik, Informatik Und Naturwissenschaften, Fachbereich Biologie, Institut Für Zell- Und Systembiologie Der Tiere, Verhaltensbiologie, Universität Hamburg, Martin-Luther-King Platz 3, 20146 Hamburg, Germany

**Keywords:** Salticids, Anti-predator behaviour, Predator-prey interaction, Mating-survival trade-off, Sensory ecology

## Abstract

Predation risk imposes fundamental trade-offs between survival and reproduction, often leading to sex- and age-specific differences in anti-predator behaviour. We investigated these trade-offs in the jumping spider *Saitis barbipes*, a species in which cryptic females and juveniles contrast with conspicuously coloured males that engage in courtship displays. Using high-quality in-focus and blurred predator images, we tested first behavioural responses and reaction distances of adult males, adult females, and juveniles. While all groups reliably recognised predator cues, adult males consistently approached stimuli more closely before showing anti-predator behaviour and frequently responded with courtship rather than defensive behaviours. In contrast, females and juveniles more often employed passive strategies such as freezing. Stimulus clarity had no effect on the type of response, whereas distance strongly influenced behavioural responses. These results indicate that predator recognition is innate, but response thresholds are context-dependent and shaped by sex, age, and reproductive role. Conspicuous adult males appear to suppress or delay anti-predator responses in favour of courtship, highlighting an evolutionary trade-off between reproductive effort and survival.

**Supplementary Information:**

The online version contains supplementary material available at 10.1186/s12983-026-00604-7.

## Introduction

Predation risk is universal and cannot be completely avoided unless an individual refrains from foraging, mating, and reproducing. This creates a fundamental trade-off: animals must constantly weigh the benefits of such activities against the increased risk of predation they entail [31]. Anti-predator strategies are manifold, ranging from cryptic colouration and mimicry to dynamic behaviours such as freezing, fleeing, or threatening displays (e.g.[9, 40, 50]). Within species, predation often affects individuals differently depending on intrinsic factors such as sex [49, 52, 62], age [33, 42], and reproductive status, for example whether females are mated or not [39]. Individual variation in anti-predator behaviour is expected because animals adjust risk-taking according to their internal state and current ecological context [31]. Moreover, animals are generally highly sensitive to cues indicating predation risk, responding systematically to both predator traits and environmental context [50].

Anti-predator responses frequently differ between males and females due to sex-specific differences in life-history strategies, reproductive investment, hormonal differences and sexual selection pressures [18, 20, 30]. Such differences in risk avoidance have been documented across various taxa, including insects [52], spiders [46], and vertebrates [6, 35].

A major driver of these differences is the unequal reproductive role of the sexes. In solitary species, males are frequently the searching sex that actively locates females [2, 14, 57]. Mate search involves increased movement activity, which may increase exposure to predators [28, 35, 46]. Where reproductive opportunities are restricted to a narrow temporal window males accept even greater risks as they cannot afford to delay or reduce their mate-searching activity [31, 34]. In contrast, species with year-round mating opportunities can afford to be more selective or cautious, spreading reproductive effort over a longer time frame and thus reducing immediate predation pressure during any single encounter [1, 6, 28, 49, 62].

In addition to finding a mate, male reproductive success frequently depends on conspicuous courtship signals, which next to females are also potentially perceived by enemies [59, 61]. Hence, signalling males underly a trade-off between investment in courtship and risk of predation [31, 34]. This trade-off can be mitigated in different ways: Male guppies reduce the conspicuousness of their ornaments under increased predation risk, [15]. Wolf spider males lower courtship intensity under predation risk if they are attractive, whereas less attractive males increase their display intensity [46].

Unlike males, females tend to prioritise survival over mating opportunities [2, 27, 31, 58]. This is especially true for females that are gravid or engage in parental care, as their current reproductive investment would be entirely lost if they fall victim to predation [31]. Such females often show increased vigilance and heightened risk avoidance -leading to reduced movement, avoidance of exposed areas, or delayed mating decisions until perceived risk levels decrease [4, 10, 12, 35],A. R. [54]. However, sex-specific risk avoidance is context-dependent and shaped by multiple ecological and physiological factors and notable exceptions to this pattern exist. For example, male snails (*Nucella lapillus)* showed greater avoidance of predation risk by reducing their activity, whereas females continued foraging to sustain reproductive investment [11].

Ontogenetic changes in morphology, cognitive ability, and experience also influence anti-predator strategies. Owing to their smaller size and reduced mobility, juveniles are likely more vulnerable to a broader range of predators [3, 7, 32, 48]. They may rely more on innate predator-avoidance responses, whereas adults refine their strategies through experience [42, 44]. Growth-survival trade-offs further shape these responses, as juveniles must balance predation risk with the need for foraging and rapid development. This trade-off can be exhibited differently depending on species-specific life-history strategies: short-lived species may tolerate higher risks early in life to maximise reproductive opportunities, whereas long-lived species may adopt more cautious behaviours during their juvenile stage to ensure future survival [34].

Jumping spiders (*Salticidae*) constitute an excellent system for studying context-dependent predator responses because of their complex visual system, predatory lifestyle, and intricate behaviour [23, 38]. They strongly rely on vision when assessing their environment, with the anterior median eyes providing exceptionally high visual acuity [5, 19, 21–23, 29, 51]. For example, *Portia labiata* uses visual information to assess prey risk and to flexibly adjust its predatory tactics (e.g. [25, 26]) as well as to visually inspect and evaluate alternative routes [53].

Jumping spiders occupy an intermediate trophic level as mesopredators, frequently encountering both prey and potential predators. In some cases, they must even distinguish between a conspecific and a threat, when, for example, encountering another jumping spider. As a distinct feature of this group, males of many jumping spider species engage in conspicuous courtship displays (e.g. [13, 17, 60], which are likely to increase predation risk [43]). However, how they balance mate attraction with predator avoidance remains poorly understood. It is unknown whether male jumping spiders exhibit reduced predator responsiveness while actively searching for mates.

Observations of immature jumping spiders showed greater activity levels and higher risk-taking tendencies in males (shorter freezing duration during a potential threatening situation) than in juvenile females [37]. Previous research has demonstrated that jumping spiders possess innate predator recognition abilities: For example, *Salticus scenicus* individuals reliably recognise static visual cues of predatory salticids and respond with a freeze-and-retreat behaviour [45]. Interestingly, and contrary to common expectations of sex-specific risk sensitivity, the response appears to be consistent across sexes, while juvenile responses have been shown to vary among jumping spider species, possibly in relation to habitat differences [41].

Due to their visually guided behaviour, even subtle manipulations of visual information can substantially alter cue reliability and the information available for decision-making in jumping spiders. Previous studies on jumping spider predator responses have used realistic, static 3D-models of jumping spider predators [41, 45]. Unambiguous stimuli may elicit uniform anti-predator responses across individuals, reducing the opportunity for among-individual differences to emerge [24, 36]. This raises the question of how jumping spiders respond when predator cues are less clear and whether behavioural responses become more variable among individuals.

*Saitis barbipes* was chosen as focal species because its courtship behaviour is conspicuous [47, 60] and strongly visually mediated (pers. obs. M.S.). Adult males frequently perform conspicuous leg-waving displays, indicating a high baseline investment in visually guided signalling. This makes the species particularly well suited for studying how visually salient mating-related behaviour interacts with predator detection and risk assessment. Moreover, while courtship behaviour in *S. barbipes* is well described [47, 60], its anti-predator responses remain largely unexplored, allowing us to test general predictions about risk sensitivity in a system with known sex-specific signalling strategies.

In our study, juvenile and adult female and male *Saitis barbipes* are tested for their behavioural responses and reaction distances to images of a large jumping spider predator presented either in-focus or blurred. Our idea was that ambiguous cues–such as a blurred image of a predator–could expose subtle differences in risk **avoidance** that may remain masked when threat levels are high and clear. We hypothesised that under such uncertainty, individuals rely more strongly on internal state and risk-taking tendencies when deciding whether and how to respond.

We predict that if predator recognition is innate and independent of life stage and sex, all spiders should respond similarly to an image of a large jumping spider. However, if predator recognition and responses are influenced by experience and/or reproductive state, we expect variation between groups–particularly under ambiguous conditions (blurry image). Accordingly, we expect females and juveniles to exhibit more cautious behaviour and to react at greater distances than adult males, regardless of stimulus clarity. Adult males, in contrast, are predicted to be more risk-prone due to their strong motivation to locate and mate with adult females and may hesitate to flee in ambiguous situations, as doing so could mean missing a potential mating opportunity.

By comparing responses across sexes, life stages, and stimulus clarity, this study aimed to assess whether anti-predator behaviour in the jumping spider *Saitis barbipes* is innate or context-dependent. These insights will enhance our understanding of how ecological and sexual selection pressures shape predator avoidance strategies in a visually guided, sexually dimorphic species with conspicuous courtship displays.

## Methods

### Spider collection and maintenance

Juvenile *S. barbipes* were collected in May 2022 and April 2023 from a deciduous forest near Osp, Slovenia, which is characterised by dense understory vegetation. Once the temperature rises above approximately 12 °C, *S. barbipes* can be found in oak leaf litter, particularly in areas where the terrain forms step-like structures, possibly remnants of former vineyards (pers. obs. MS).

Each spider was housed individually in a plastic container (10 × 6 × 4 cm) containing a piece of paper towel, wood shavings and a piece of folded cardboard that served as a shelter and provided structural complexity. Mesh-covered openings in the longer sides of the containers ensured adequate ventilation. Opaque partitions separated all container, to avoid visual contact to other individuals. Illumination was supplied by daylight lamps (6500 K ABSOLUTE SERIES™ LED Flexible Strip, Waveform Lighting LLC, Vancouver, USA) and supplementary UV illumination at 365 nm (realUV™ LED Strip Lights, Waveform Lighting LLC, Vancouver, USA) and 395 nm (DT5UV, Onforu Technology Ltd, Shenzhen, China) to resemble the spectral composition of natural daylight and was set to a 15:9h light–dark cycle. The room temperature averaged 24 °C, and relative humidity was maintained at approximately 60%. Containers were sprayed with water daily to sustain humidity levels. Spiders were fed twice weekly with *Drosophila sp.,* always in surplus of their feeding requirements. Both, adults and smaller juveniles were able to easily catch this type of prey. Individuals were checked daily four moulting, allowing the date of maturation to be recorded. In 2022 juveniles were reared to adulthood to test them as adults, and in 2023 juvenile spiders were tested before becoming sexually mature. Tested adults (n = 61) had a total body length of 4–6 mm and juveniles (n = 49) 3–6 mm. Unless close to their final moult into sexual maturity, juvenile females and males are indistinguishable in appearance, and their sex could not be reliably determined until they reached adulthood. Adults were tested within 50 days after their final moult (median: 7 days), and juveniles between 55 and 1 day before their final moult (median: 7 days before adulthood). In total, 30 adult females, 23 juvenile females, 31 adult males and 14 juvenile males were tested along with 12 juveniles that did not reach adulthood and therefore could not be assigned to a sex (Table 1).

**Table 1:** Summary of the number of spiders tested per treatment. Juvenile individuals that could not be sexed (last row) were retained in the dataset for analysis of total occurrence of behavioural responses. Number in parentheses refer to individuals that responded to the stimulus

Females	In-focus	Slightly blurry	Very blurry
Adult	30 (30)	29 (28)	25 (1)
Juvenile	23 (23)	18 (17)	19 (2)

Males	In-focus	Slightly blurry	Very blurry
Adult	29 (26)	30 (29)	31 (3)
Juvenile	13 (12)	13(12)	14 (1)

Not sexed	In-focus	Slightly blurry	Very blurry
Juvenile	12 (11)	11 (11)	12 (1)

### Stimulus

**Fig. 1:**
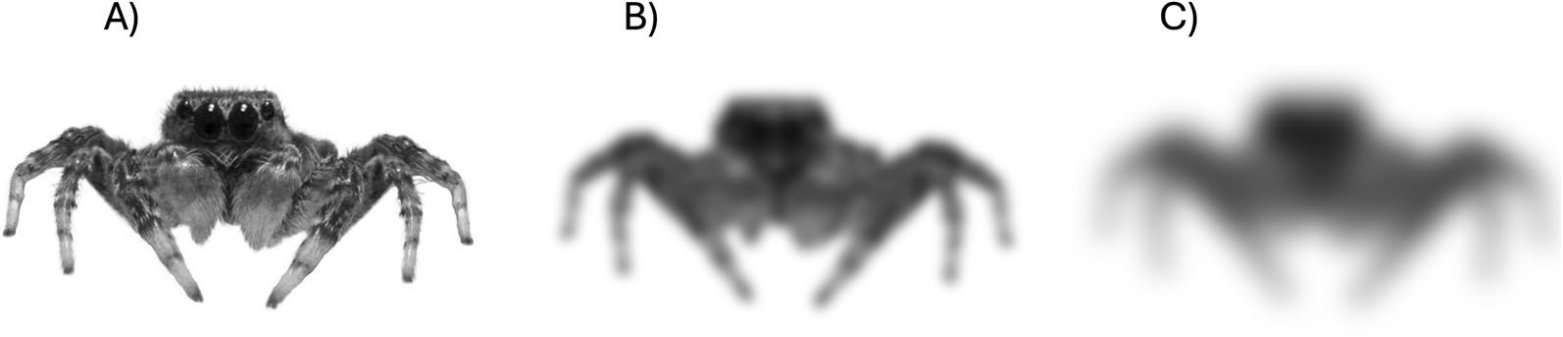
Visual predator stimuli used in the experiment. **A** Sharp image based on an adult *Marpissa muscosa*, **B** moderately blurred version (Gaussian blur, 5 pixels), and **C** heavily blurred version (Gaussian blur, 10 pixels). All stimuli were bilaterally symmetric

Predator responses were tested with black and white images of spiders printed on white paper. Standard photo printing methods create small dots that are probably visible to spiders. To avoid this kind of pixelation and ensure a more naturalistic representation, all the images were developed using high-quality Ilford paper through a traditional photographic process. The stimulus was based on a high-resolution black and white photograph of an adult *Marpissa muscosa.* We chose this species because it is widespread across Europe and co-occurs with *Saitis barbipes* in the same habitat*.* However, our aim was not to test a response to a specific species but using feature characteristics for a jumping spider, in particular the distinct eye arrangement and leg posture*.* To control for potential lateral bias, the image was digitally adjusted to achieve bilateral symmetry. The final stimulus measured 17 mm in width (distance between outer leg tips) and 8 mm in height (from front leg tip to head) (Fig. 1). In comparison, adult *Saitis barbipes* have an average body width of 3 mm (adult female body length: 5.3 ± 0.4 mm, adult male: 4.6 ± 0.5 mm, juv. female: 4.4 ± 1.2 mm, juv. male: 3.8 ± 1.1 mm). The stimulus thus represents a comparatively large jumping spider relative to our test species.

To test whether the spiders respond to an image at all, an image with a large jumping spider was tested against an image with a circle of the same size (unpublished data). All spiders showed a clear behavioural response to the image and we started the main trials in which the spiders were presented with three types of images: a focused image of a larger jumping spider, an image with Gaussian blur (5 pixels) and a heavily blurred image (10 pixels) (Fig. 1).

### Experimental setup

**Fig. 2:**
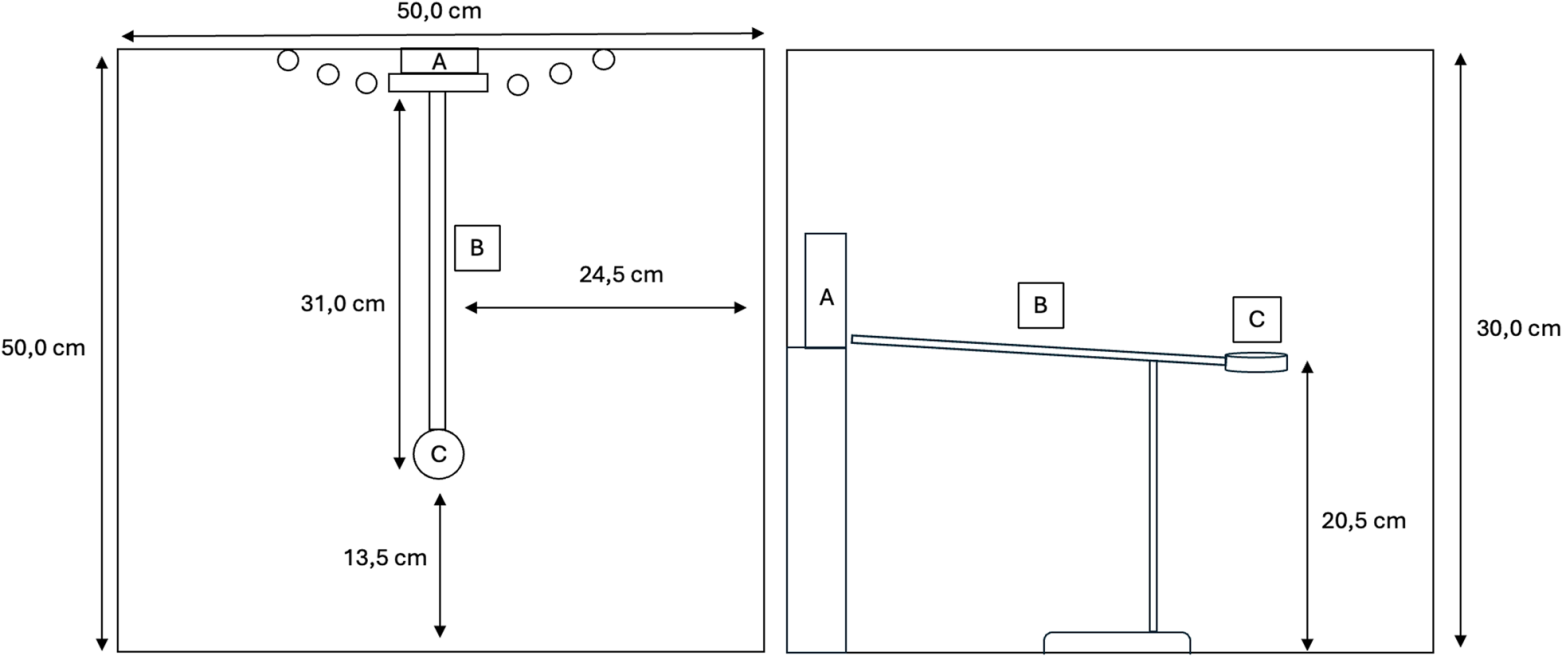
Schematic of the experimental setup, shown from top-down view (left) and side view (right). The setup includes the stimulus (**A**), the 3D-printed ramp (**B**), and the starting platform **C**. The dimensions indicate the distances between components and the overall box size

The experiments were conducted in a wooden box (50 × 50 × 30 cm) to shield the spiders from the external movements of the observer, minimising distractions. Trials were recorded using an iPhone mounted on a holder outside the arena, filming the ramp from a lateral and slightly elevated angle. The setup consisted of a narrow 3D-printed ramp with a small, circular platform at the end, leading to the visual stimulus (Fig. 2). Wooden sticks were positioned on both sides of the stimulus to create a sense of depth and to encourage the spiders to approach the image [45]. A slight incline of approximately 6 degrees also encouraged the spiders to move upwards toward the presented image, while keeping the stimulus fully visible. The ramp measured 310 mm in length and had a width of 10 mm. Precise distance measurements during video analysis were facilitated by small markers every 5 mm along the ramp (Fig. 2). The ramp height was set to 20 cm, based on prior observations showing that spiders tend to jump directly to the ground from lower heights. At 20 cm or above, they rarely do so (pers. observations).

A trial started by placing the spider gently onto the small platform at the end of the ramp, opposite the stimulus, which was already positioned. Video recording started before the spider was introduced to ensure that no reaction was missed. Each individual was then given five minutes to move onto the ramp. If it failed to do so, the trial was terminated, and the spider was tested again on another day.

Once on the ramp, each spider was given a maximum of ten minutes to react or reaching the image stimulus. *S. barbipes* show a clear preference for moving on top of structures and are rarely observed to walk upside down underneath structures (pers. obs. J.A., M.S.). This was very helpful, as most spiders did not even attempt to move below the ramp, and a very thin layer of Vaseline was sufficient to make the underside of the ramp even less attractive. The Vaseline coating was kept to a minimum to avoid potential effects on the spiders. They could still cling to the underside of the ramp but usually quickly returned to the upper surface.

After each trial, the ramp was cleaned with 70% isopropyl alcohol to remove any potential chemical cues such as pheromones or silk residues that could influence subsequent trials. However, preliminary observations revealed that spiders were more likely to jump immediately after encountering the freshly alcohol-cleaned surface, even when it appeared dry. To counteract this, the ramp was additionally rinsed with deionised water and dried with tissue paper before the next trial, which apparently solved this issue. Room temperature was measured and included as control variable, as it can influence behaviour and could not be fully controlled in the experimental room.

In order to minimise potential learning effects, as well as immediate carry-over and habituation effects, all spiders were given a break of at least one day between trials. The mean inter-trial interval was 3.2 ± 1.6 days, except one outlier with 27 days between trials. Additionally, the order of trials was included as a control variable to account for potential effects.

We aimed to test each spider three times, once with each image, with the order of treatments randomised to prevent sequence effects. However, some individuals did not participate in all treatments due to death, moulting into adulthood, or egg laying.

### Video analyses

Videos were analysed using BORIS software [16] to track behavioural responses and reaction distances. Each time a distinct behaviour was observed, it was recorded and the spider’s position (distance to stimulus) was noted. We focused on behaviours that occurred after the spider oriented towards the stimulus, excluding routine movement such as walking, scanning, or pedipalp motion. Thus, our observations were naturally biased towards behaviours that could be clearly interpreted as reactions to the stimulus. The reactions were classified into different categories (Table 2). We were able to observe and classify two different types of responses: male courtship displays and behaviours that we interpreted as anti-predator responses.

**Tab 2:** Classification and description of behavioural responses

Type of behaviour	Description
Freezing	Spiders are oriented towards the stimulus image, come to a complete stop and remain entirely motionless for at least 10s. Even the pedipalps, which typically exhibit subtle movements even when the spider normally stops, become completely still
Directional change	Spiders are oriented towards the stimulus image, stop, and then turn around to move the ramp down in the opposite direction
Retreat	Spiders are oriented towards the image, stop, and slowly move backwards This behaviour was often shown after a period of freezing behaviour
Hiding	After orienting towards the stimulus image, spiders quickly move under the ramp to hide
Jumping down	Spiders are oriented towards the image and quickly decide to leave the ramp by jumping down
Courtship display	Male spiders, after orienting towards the stimulus image, lift their third pair of legs (“courtship1/ leg waving”, Fig. 3) Some individuals also perform a distinctive “leaning” behaviour [60], referred to as “courtship 2”/ “leaning”, which is typically observed before courtship leg-drumming (Fig. 3)

**Fig. 3:**
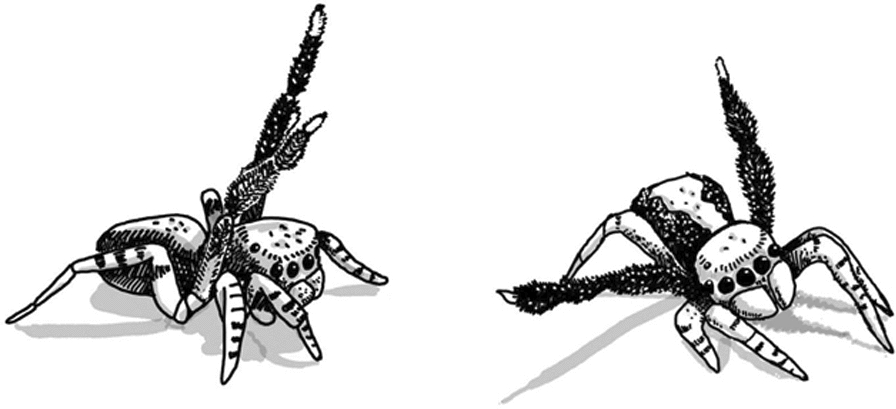
Male *S. barbipes* is lifting his third pair of legs (left) and shows a typical courtship behaviour by “leaning” to one side (right). Illustration by M.S.

### Statistical analysis

All statistical analyses were performed in R Studio (version 4.5.1 “Great Square Root”).

Analyses focused on two response types: (1) first reaction (the initial response of each individual that we could observe) and (2) first anti-predator reaction (first reaction that was not courtship behaviour). This allowed us to compare initial responses and responses distances—which may indicate cautiousness or attentiveness—with the distances at which spiders decided to avoid the situation, providing insight into the risk-taking tendency.

Because most individuals did not react to the very blurry image, we restricted all analyses of behavioural categories and reaction distance to trials with the in-focus and slightly blurred image (hereafter “in-focus” and “blurry”).

For each data set, the type of behaviour was used as the response variable and analysed using Bayesian categorical models fitted with the *brms* package. Fixed predictors included treatment (in-focus/ blurry), sex (male, female), age group (adult, juvenile), room temperature (continuous, range 18.4–28.3 °C) and trial order (1/2/3). A random intercept for individual ID accounted for repeated measures. We used weakly informative priors: Normal (0, 2) for fixed effects, Student-t (3, 0, 2.5) for intercepts, and Exponential (1) for random-effect standard deviations. All analyses were performed using the *cmdstanr* backend with four chains (4000 iterations each, 1000 warm-up). Model convergence was confirmed by R-hat = 1 and sample sizes > 1000. Model structure was selected using Bayes factor model comparison based on bridge sampling. Predictors improving model fit were retained: age group (adult/ juvenile), sex (male / female), treatment (in-focus/ blurry), room temperature, and order of stimulus presentation. Interactions among age group, sex, and treatment were tested and simplified when unsupported. Posterior predictions were extracted with *posterior_epred()* allowing us to calculate contrasts between factor levels.

In addition, linear mixed-effects models (*lme4, lmerTest*) were used to analyse the distance at first reaction and the distance at first anti-predator behaviour, with sex, age group, and treatment as fixed factors and individual ID as random effect. Model simplification was performed by sequentially removing non-significant interaction terms and comparing models via likelihood ratio tests. Significant pairwise contrasts were further explored using *emmeans* with Tukey adjustment.

Differences among experimental groups and treatments explained most of the observed variation in behavioural responses. Although model comparison indicated that both room temperature and trial order improved model fit, the corresponding parameter estimates suggested only weak and partly inconsistent effects. They were mainly retained as control factors.

## Results

A total of 95.3% (102 of 107) of the tested *S. barbipes* reacted to the in-focus image, and 96.0% (97 of 101) reacted to the slightly blurred image. In contrast, only 7.9% (8 of 101) reacted to the very blurry image.

**Fig. 4:**
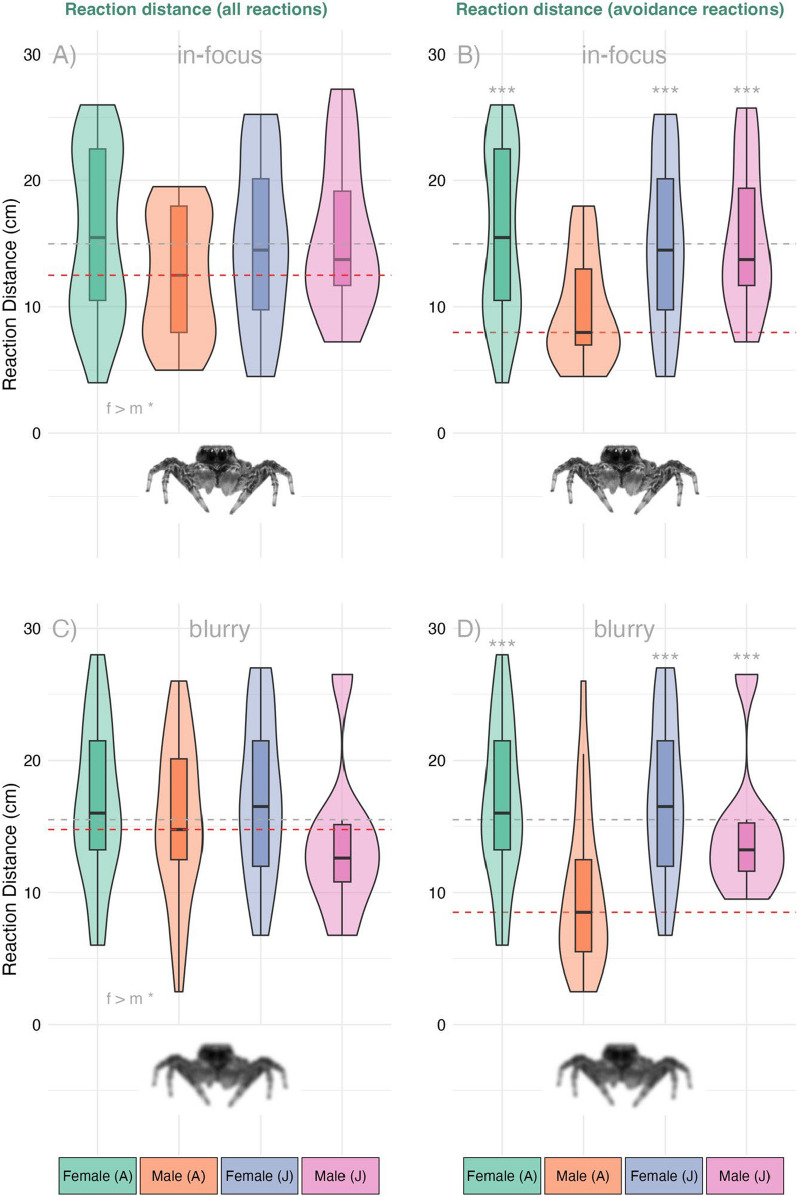
Reaction distances of adult (**A**) and juvenile (**J**) female and male *S. barbipes* towards a predator stimulus. Panels (**A**) and (**B**) show responses to the in-focus image, whereas panels (**C**) and (**D**) show responses to the blurry image. Panels on the left (**A**, **C**) display distances for all reactions, whereas panels on the right (**B**, **D**) show distances at which spiders exhibited a predator avoidance response (excluding courtship displays). The grey dashed lines indicate the median distance of juvenile males and females as well as adult females, whereas red dashed lines show the median distance of adult males for comparison. Asterisks in **B**) and **D**) indicate significant differences in reaction relative to adult males for the respective group

There were sex differences in the distance at which individuals first reacted to the stimulus, with males approaching the stimulus further than females (estimate = –1.91 ± 0.96 SE, *t* = –1.99, *p* = 0.049, Fig. 4). No difference was found between juveniles and adults, when sex was not considered as a factor (estimate = 0.32 ± 0.99 SE, *t* = 0.33, *p* = 0.74).

When restricting the analysis to anti-predator reactions only, the distance at which individuals responded was significantly influenced by the interaction between sex and age (*sex* × *age group*: *t* = 2.78, *p* = 0.007). Adult males initiated predator avoidance around 6 cm closer to the stimulus than adult females (estimate = -6.36 ± 1.17 SE, *p* < 0.001, Fig. 4), juvenile females (estimate = -6.15 ± 1.28 SE, *p* < 0.001, Fig. 4) and juvenile males (estimate = -5.46 ± 1.63 SE, *p* = 0.006, Fig. 4). Neither treatment nor age group alone had a significant effect on predator avoidance distance (Fig. 4).

**Fig. 5:**
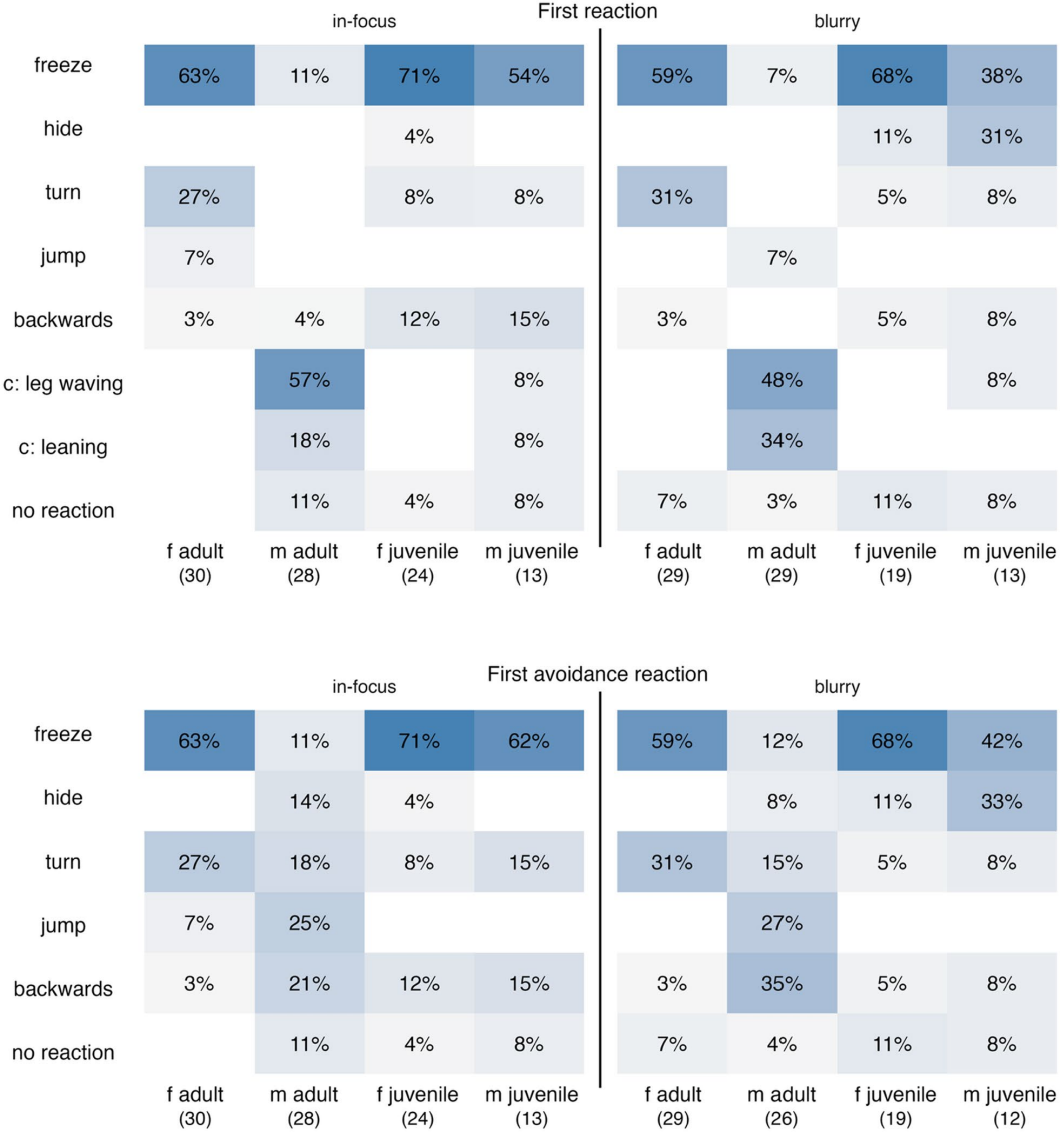
Proportion of initial behavioural responses displayed by *Saitis barbipes* when exposed to visual stimuli. The Left panels show reactions to the in-focus stimulus the right panels show reactions to the blurry stimulus. Top: first observed behaviour; bottom: first anti-predator reaction. The percentages indicate the proportion of individuals exhibiting a given behaviour within each sex and age group. The total sample sizes are displayed below the groups

The type of first reactions of the spiders to the in-focus and blurry image were largely similar. Also, most behaviours did not differ significantly between adults and juveniles. Where differences occurred, they were primarily driven by adult males, which showed a significantly higher probability of performing courtship behaviour and a markedly lower probability of freezing and turning compared to other groups (Fig. 5).

Anti-predator reactions also did not differ between treatments or age groups. However, adult males showed a markedly lower probability of *freezing* and a higher probability of hiding and jumping compared to other groups (Fig. 5). Complete statistical results are provided as supplementary material (Suppl. 1).

**Fig. 6:**
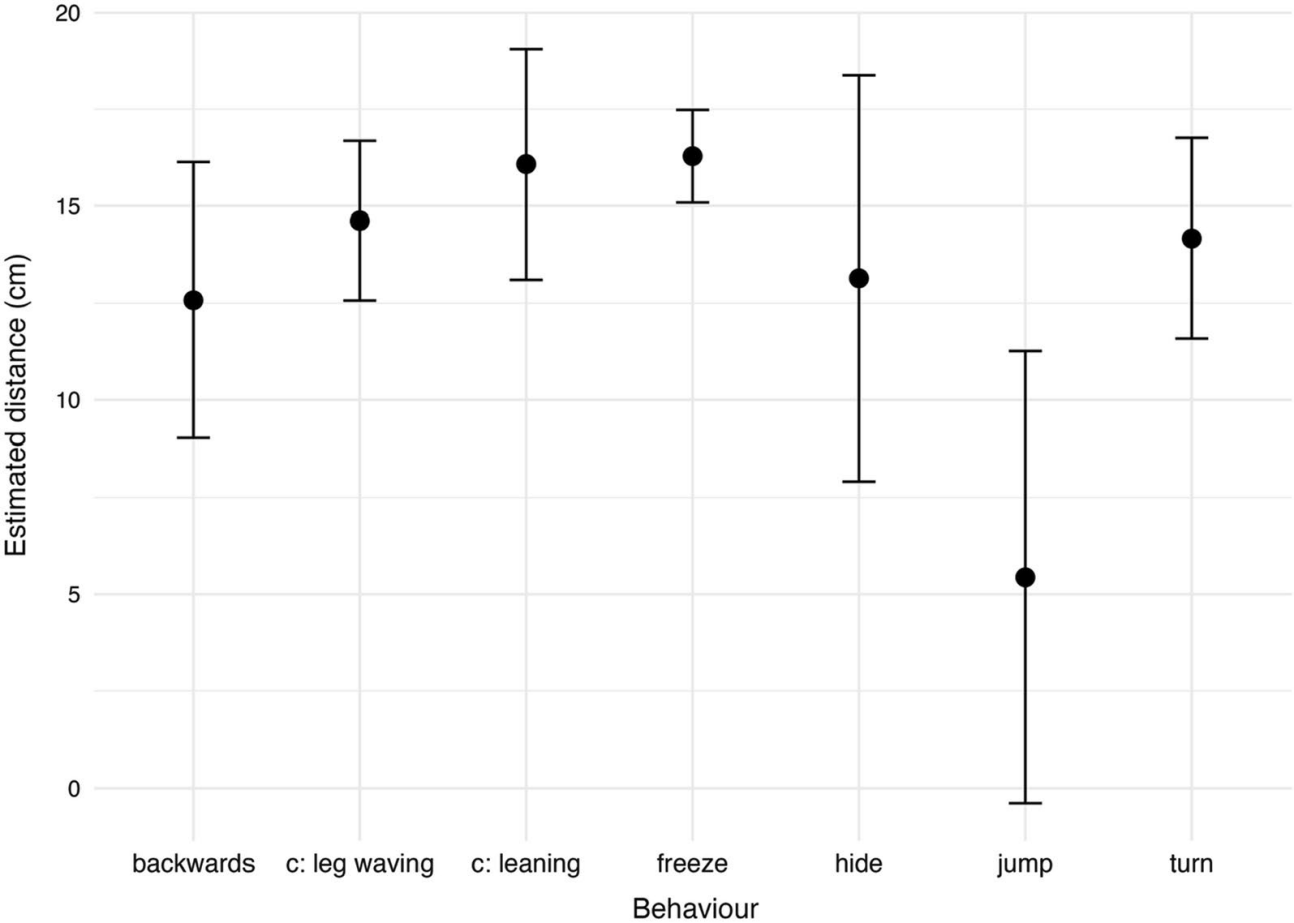
Estimated distances (cm) at which *Saitis barbipes* first displayed a given anti-predator behaviour in response to the visual stimulus. Model estimates (points) with 95% confidence intervals from a linear mixed model including behaviour as a fixed effect and individual identity as a random effect. Behaviours are averaged across sex and age groups

The type of anti-predator behaviour initially displayed varied with the spider’s position on the ramp, i.e. its distance from the image at the moment it noticed the stimulus. (Fig. 6): Hiding (ß = − 0.174, 95% CI [− 0.370, − 0.017]) and jumping down from the ramp (ß = − 0.716, 95% CI [− 1.640, − 0.254]) was much more likely in closer distance whereas the probability of freezing was greater when individuals reacted further away from the stimulus (ß = 0.164, 95% CI [0.065, 0.284]).

## Discussion

Exposure to the predator stimulus elicited a variety of behavioural responses across individuals and groups. Contrary to our predictions, reaction distance or behaviour did not differ between the in-focus and the blurry predator images. These results suggest that spiders either did not discriminate between stimulus clarity, or that both provided sufficient visual information to elicit a behavioural response. The outline of a large jumping spider alone seems to be sufficient to provoke anti-predator behaviour confirming previous findings of innate predator recognition in jumping spiders (e.g. [45]) and adding that jumping spiders also respond to two-dimensional predator cues. The low response rate to the heavily blurred stimulus suggests that visual details are crucial to stimulus recognition, and that spiders do not simply react to any dark object at the end of the ramp in our experiment. The overall median reaction distance of 14.5 cm is greater than suggested by a previous study on jumping spider responses to their own mirror image, in which most species reacted at a distance below 10 cm [22]. However, that study considered only male-male displays (raised legs) and ignored other reactions. In addition, our stimulus was considerably larger, which may explain the earlier reactions observed here.

The response distance was broadly similar across all groups, indicating that all spiders recognised the stimulus at a similar distance. Male first reactions occurred at slightly shorter distances than those of females. In contrast, adult males stand out by moving closer to the stimulus before initiating an anti-predator response. This suggests sex- and age-related differences in risk perception and decision thresholds. Adult males may accept greater risk to maximise their mating opportunities, while females, especially adults, are more cautious. These findings support the notion that females prioritise risk avoidance [4, 11] and are consistent with evidence from other taxa showing that risk-management strategies are flexible and shaped by sex, condition and life-history trade-offs [28, 37, 46].

In our experiments, adult males often displayed courtship behaviour first, a rather conspicuous behaviour, to draw the attention of potential mates. This pattern suggests that males downregulate anti-predator responses in favour of securing mating opportunities, reflecting a risk-reward trade-off. Contrary to our expectation that visual ambiguity would modulate male behaviour, males courted consistently, even under clear predatory threat, indicating that adult males may prioritise mating opportunity over caution irrespective of threat clarity.

It is possible that males misidentified the stimulus as conspecific female rather than a threat. Such a misinterpretation is particularly striking given that the depicted spider was not only significantly larger than a conspecific female, but also clearly male, making a conspecific mating target highly unlikely. However, similar observations were reported from multiple *Habronnatus* species, where males frequently engage in courting heterospecifics thereby risking predation. A strong selection pressure to use each mating opportunity might be responsible for limited discrimination abilities (L. A. [56]). Similar trade-offs have been observed in *Hygrolycosa rubrofasciata*, where males that increased courtship signalling and mate searching achieved higher mating success, but also suffered a greater risk of predation [28].

Particularly in encounters with fast-striking predators, even small differences in reaction distance can affect survival. Although we cannot be sure whether the 6 cm difference in males closer to the potential predator compared with the other groups is ecologically relevant under natural conditions, it is likely that males face an increased risk of predation when suppressing or delaying escape responses in favour of courtship attempts. Male *S. barbipes* anti-predator responses may be delayed or suppressed during courtship or mate searching, reflecting a general evolutionary trade-off between risk-avoidance and mating effort [34].

The finding that males showed leg-waving towards a predator-like image could also mean that the behaviour may not be limited to courtship. In *Habronnatus*, leg-waving combined with conspicuous dorsal patterns (similar to those of *S. barbipes* males) has been proposed to mimic the antennal movements of hymenopterans, potentially deceiving predators (L. A. [55]). Experimental tests of this mimicry hypothesis revealed that spiders with male-like dorsal patterns were attacked more frequently than those with cryptic female-like patterns, regardless of sex and males were captured more often and sooner than females regardless of their dorsal pattern [8] contradicting the protective function of mimicry in this context. Leg-waving in *S. barbipes* was often followed by leaning behaviour, which is only observed in a courtship context (pers. observation). Whether leaning contributes to predator distraction remains unclear. However, based on our observations, leg-waving of males likely reflects a courtship response triggered by misidentification of the predator stimulus.

When discussing different anti-preator strategies, an additional factor to consider is colouration: Male *Saitis barbipes* appear—at least to the human visual system—more conspicuously coloured (iridescent blue-green eyes, a light-red frontal band, and red third legs transitioning into black with contrasting white tips), whereas females and juveniles are cryptic (brown-grey body with mottled patterning, lacking conspicuous ornamentation or bright colour contrasts). Freezing reduces movement cues used by predators. For cryptically coloured individuals, like females and juveniles, freezing may be an effective anti-predator strategy. In contrast, conspicuous males may benefit less from freezing, especially when they already draw attention through movement (e.g. courtship), making active escape behaviours more effective and potentially explaining their lower freezing rates.

The response type also seems to depend on reaction distance. At larger distances, spiders are more likely to freeze and withdraw, whereas at closer distances to the stimulus, they tend to employ active escape strategies such as hiding or jumping down the ramp. This pattern indicates a graded anti-predator strategy rather than a single fixed response.

Although behavioural differences in anti-predator behaviour between groups were observed, limited subgroup sample sizes make it unclear whether these patterns reflect true age- and sex-specific response or sampling variability.

While we interpret the pronounced male responses as reflecting a trade-off between mating effort and predator avoidance, alternative explanations should be considered. Male-specific patterns may partly arise from attentional or motivational biases associated with courtship, potentially delaying threat processing rather than reflecting increased risk tolerance per se. In addition, the simplified experimental context and the use of static visual stimuli may have constrained the expression of natural escape strategies*.* Future studies could investigate whether the observed sex-specific responses are consistent under more naturalistic conditions, including variable backgrounds, light conditions, moving predator stimuli or distractions. It would also be valuable to test whether males alter their trade-off decisions under increased perceived predation risk, for example by adding additional predator cues such as chemical traces. Finally, investigating reaction latency could provide additional insight into attention and predator detection abilities.

In summary, predator recognition in *S. barbipes* appears innate, whereas subsequent behavioural responses are flexible and context-dependent. The graded responses observed here were associated with stimulus distance and varied across sex and life stage, reflecting dynamic survival- reproduction trade-offs. Our study therefore underscores the importance of considering perception, motivation, and life-history context when interpreting predator–prey interactions and highlights that mating-related motivations can substantially modulate risk-sensitive behaviour.

## Supplementary Information

Additional file 1 (XLSX 23 KB)

Additional file 2 (CSV 48 KB)

## Data Availability

All data generated or analysed during this study are included in this published article [and its supplementary information files].
